# Diversity and Population Overlap between Avian and Human Escherichia coli Belonging to Sequence Type 95

**DOI:** 10.1128/mSphere.00333-18

**Published:** 2019-01-16

**Authors:** Steffen L. Jørgensen, Marc Stegger, Eglé Kudirkiene, Berit Lilje, Louise L. Poulsen, Troels Ronco, Teresa Pires Dos Santos, Kristoffer Kiil, Magne Bisgaard, Karl Pedersen, Lisa K. Nolan, Lance B. Price, Rikke H. Olsen, Paal S. Andersen, Henrik Christensen

**Affiliations:** a Department of Veterinary and Animal Sciences, University of Copenhagen, Frederiksberg, Denmark; b Department of Bacteria, Parasites and Fungi, Statens Serum Institut, Copenhagen, Denmark; c National Veterinary Institute, Technical University of Denmark, Frederiksberg, Denmark; d Bisgaard Consulting, Viby Sjælland, Denmark; e Department of Infectious Diseases, College of Veterinary Medicine, University of Georgia, Athens, Georgia, USA; f Milken Institute School of Public Health, George Washington University, Washington, DC, USA; U.S. Centers for Disease Control and Prevention

**Keywords:** *E. coli*, genomics, pathogenicity, sequencing, zoonosis

## Abstract

Avian-pathogenic Escherichia coli (APEC) is a subgroup of extraintestinal pathogenic E. coli (ExPEC) presumed to be zoonotic and to represent an external reservoir for extraintestinal infections in humans, including uropathogenic E. coli (UPEC) causing urinary tract infections. Comparative genomics has previously been applied to investigate whether APEC and human ExPEC are distinct entities. Even so, whole-genome-based studies are limited, and large-scale comparisons focused on single sequence types (STs) are not available yet. In this study, comparative genomic analysis was performed on 323 APEC and human ExPEC genomes belonging to sequence type 95 (ST95) to investigate whether APEC and human ExPEC are distinct entities. Our study showed that APEC of ST95 did not constitute a unique ExPEC branch and was genetically diverse. A large genetic overlap between APEC and certain human ExPEC was observed, with APEC located on multiple branches together with closely related human ExPEC, including nearly identical APEC and human ExPEC. These results illustrate that certain ExPEC clones may indeed have the potential to cause infection in both poultry and humans. Previously described ExPEC-associated genes were found to be encoded on ColV plasmids. These virulence-associated plasmids seem to be crucial for ExPEC strains to cause avian colibacillosis and are strongly associated with strains of the mixed APEC/human ExPEC clusters. The phylogenetic analysis revealed two distinct branches consisting of exclusively closely related human ExPEC which did not carry the virulence-associated plasmids, emphasizing a lower avian virulence potential of human ExPEC in relation to an avian host.

**IMPORTANCE** APEC causes a range of infections in poultry, collectively called colibacillosis, and is the leading cause of mortality and is associated with major economic significance in the poultry industry. A growing number of studies have suggested APEC as an external reservoir of human ExPEC, including UPEC, which is a reservoir. ExPEC belonging to ST95 is considered one of the most important pathogens in both poultry and humans. This study is the first in-depth whole-genome-based comparison of ST95 E. coli which investigates both the core genomes as well as the accessory genomes of avian and human ExPEC. We demonstrated that multiple lineages of ExPEC belonging to ST95 exist, of which the majority may cause infection in humans, while only part of the ST95 cluster seem to be avian pathogenic. These findings further support the idea that urinary tract infections may be a zoonotic infection.

## Abstract (precis)

APEC causes a range of infections in poultry, collectively called colibacillosis, and is the leading cause of mortality and is associated with major economic significance in the poultry industry. A growing number of studies have suggested APEC as an external reservoir of human ExPEC, including UPEC, which is a reservoir. ExPEC belonging to ST95 is considered one of the most important pathogens in both poultry and humans. This study is the first in-depth whole-genome-based comparison of ST95 E. coli which investigates both the core genomes as well as the accessory genomes of avian and human ExPEC. We demonstrated that multiple lineages of ExPEC belonging to ST95 exist, of which the majority may cause infection in humans, while only part of the ST95 cluster seem to be avian pathogenic. These findings further support the idea that urinary tract infections may be a zoonotic infection.

## INTRODUCTION

Escherichia coli is an important pathogen of both poultry and humans and may cause both intestinal and extraintestinal infections (1, 2). E. coli bacteria that are causing extraintestinal infections are known as extraintestinal pathogenic E. coli (ExPEC). ExPEC include uropathogenic E. coli (UPEC), which is the primary cause of urinary tract infections in humans,and neonatal meningitis E. coli (NMEC), which is an important cause of bacterial meningitis in human neonates (1–3). Avian-pathogenic E. coli (APEC) is an ExPEC that causes a range of infections in poultry collectively known as avian colibacillosis, which is one of the leading causes of infection in the poultry industry throughout the world (4–6). Furthermore, a growing number of infections caused by antimicrobial-resistant human ExPEC strains has complicated treatment of extraintestinal infections (2, 3).

In humans, the main reservoir of ExPEC is the host’s own intestinal tract, but a number of recent studies have suggested that the external reservoir of ExPEC may be of animal origin, including broilers, where the ExPEC is transmitted via the animal food chain (2, 7, 8). These speculations have spawned a large number of comparative studies that have demonstrated an overlap in serogroups, sequence types (STs) and virulence-associated genes, which have previously been suggested to be used for classification or even as a diagnostic tool of APEC and human ExPEC (5, 9, 10). Studies have demonstrated that some human ExPEC strains cause disease in chicken infection models, and some APEC strains cause disease in mammalian models of human disease (7, 11–13). Furthermore, identical or nearly identical pulsed-field gel electrophoresis (PFGE) profiles have been identified in specific serotype-phylogroup-sequence type groups from human extraintestinal infections and avian colibacillosis (14).

ExPEC strains belonging to ST95 have been prevalent causes of human disease (15, 16). However, strains of ST95 have also been found to be among the predominant STs causing avian colibacillosis (8, 17–19). Studies have demonstrated a close relationship between ST95 APEC and human ExPEC isolates belonging to the same sero-phylo groups, including O1/O2/O18/O45:H7-B2. Certain APEC and human ExPEC have also been found with highly similar PFGE profiles (11, 14, 20). Additionally, comparative whole-genome sequencing-based studies have demonstrated a close relationship between the ST95 APEC O1 type strain and human ExPEC strains (21–23). However, it is evident that to accurately investigate the genetic properties of APEC and human ExPEC, comparative genomic analyses of a large and diverse collection from both humans and birds are required. A better understanding of ExPEC isolates belonging to ST95 may reveal whether certain sublineages have adapted to constitute unique host-specific “subpathotypes.”

The present study aimed to compare the genetic composition of 32 APEC and 291 human ExPEC genome sequenced isolates belonging to ST95. Using these data, we investigated whether distinct genomic features of ST95 strains from different hosts exist and how these may provide insight into host adaptation as it pertains to ExPEC.

## RESULTS

**FIG 1:**
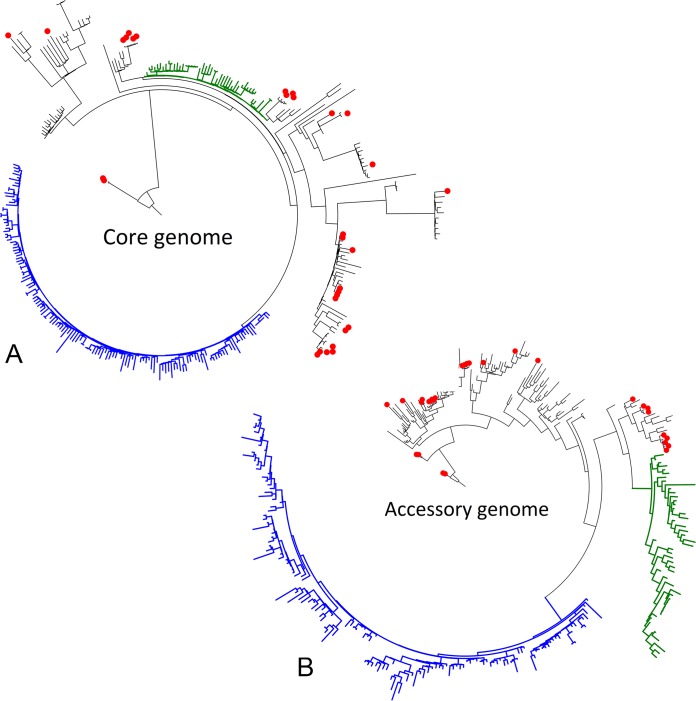
Rooted phylogenetic trees of the core genome (A) and accessory genome (B) of 323 genomes in the study. APEC genomes are marked with red dots on the branch ends. Subclades consisting exclusively of human ExPEC were identified in the core and accessory phylogenies and indicated by colored branches. The human ExPEC/HExPEC-1 subclade (blue) was distinct in both the core and accessory genome phylogenies. The HExPEC-2 subclade (green) was identified in the accessory-genome-derived tree, while a less distinct clustering was identified in the core genome phylogeny. Branches with mixes of both APEC and human ExPEC were designated A/E-PEC branches.

The initial pan-genomic analysis identified 16,786 genes, of which 3,789 belonged to the core genome. Diversity within the core genome of the APEC and human ExPEC strains were characterized by 17,468 single nucleotide polymorphisms (SNPs), while differences in the accessory genome were based on the presence or absence of the genes from the analysis of the pan-genome. A purged SNP phylogeny was constructed based on the identified SNPs with recombination regions removed (Fig. 1A). Meanwhile, presence or absence of the genes in the accessory genome was used to generate an accessory-genome-derived tree (Fig. 1B). Both trees demonstrated similar clustering, with APEC strains located on several different branches mixed with human ExPEC strains. Analysis of the SNPs by univariate and multivariate analysis could not identify single SNPs or a combination of SNPs which explicitly defined either APEC or human ExPEC. No single gene or combination of genes could separate and describe either the APEC or human ExPEC genome.

Thus, APEC belonging to ST95 did not constitute a unique branch, and it was not associated with any SNP(s) or accessory genome features. APEC was located on multiple branches of mixed APEC and human ExPEC strains in both the SNP phylogeny and the accessory-gene-derived tree (Fig. 1A and B), demonstrating a great overall genetic overlap with certain human ExPEC strains, including closely related APEC and human ExPEC strains. These branches of mixed APEC and human ExPEC strains were designated A/E-PEC. Three minor A/E-PEC clades of closely related strains, which included both APEC and human ExPEC, were identified in both the SNP phylogeny and the accessory-gene-derived tree (clades 1 to 3) (see Fig. S1A and B in the supplemental material). SNP analysis indicated that genomes within the clades differed by no more than 32 SNPs (clade 1), 57 SNPs (clade 2), or 30 SNPs (clade 3), which further underlined the close relationship of certain APEC and human ExPEC strains. The associated metadata regarding the year of sampling and country of isolation presented no indication of the clinical relationship between the isolates but rather indicated highly dispersed clones.

10.1128/mSphere.00333-18.1 FIG S1 Supplementary phylogenetic trees of the core genome (A) and accessory genome (B) of 323 genomes in the study. APEC genomes are marked with red dots on the branch ends. HExPEC subclades are indicated by colored branches as follows: HExPEC-1 subclade is shown in blue, and HExPEC-2 subclade is shown in green. Surrounding symbols indicate FimH type (squares), isolation country (triangles), and isolation year (circles). Download FIG S1, PDF file, 1.4 MB. Copyright © 2019 Jørgensen et al. 2019 Jørgensen et al. This content is distributed under the terms of the Creative Commons Attribution 4.0 International license.

Two clusters exclusively containing human ExPEC strains were identified in the two phylogenies. A large cluster consisting solely of closely related human ExPEC strains (*n* = 145) was observed in both the SNP phylogeny and accessory-genome-derived tree and designated human ExPEC 1 (HExPEC-1) and represented strains over a period of 67 years from multiple countrie sbut with an overrepresentation of strains collected in the United States (USA) in 2012 (62%) (Fig. 1A and B). An additional human ExPEC cluster (HExPEC-2) was identified in the accessory-genome-derived tree (*n* = 48) (Fig. 1A). Strains of the HExPEC-2 cluster showed a distinct separate branch in the accessory-genome-derived tree; however, the cluster did not demonstrate the same distinct separation as in the SNP-based phylogeny (Fig. 1B). Almost all strains in the HExPEC-2 cluster (94%) were collected in North America over a period of 38 years and also demonstrated an overrepresentation of strains from the USA in 2012 (46%).

Multivariate analysis was performed to distinguish the A/E-PEC, HExPEC-1, and HExPEC-2. The analysis identified two canonical but nonsynonymous SNPs both found in the *nadR* gene, which separated the large HExPEC-1 cluster from the remaining strains (Fig. 1). Furthermore, two presence/absence gene combinations were identified that could characterize and separate the HExPEC-1 cluster from the remaining genomes. The first gene combination included the copresence of *yjjW* and an identified gene labeled gene3477 (see Table S1 in the supplemental material), but an absence of *yaeP* (Table 1, gene profile A/B). Gene3477 encoded a putative transposase (GenBank accession no. CAV01995.1). The second gene combination reported the absence of the six genes belonging to the *fec* protein operon (*fecA*2, *fecB*, *fecC*, *fecD*, *fecE*, and *fecI*) and presence of putative gene8134, which could not be further characterized by either Prokka, NCBI BLAST, or UniProt (Table 1, gene profile C) (Table S1). The HExPEC-2 was described by the absence of both the *fec* operon and gene8134 (Table 1, gene profile E). Furthermore, the HExPEC-2 could be identified by the copresence of *cutC* and *cutD* (Table 1, gene profile F). The mixed A/E-PEC clusters could be separated from the two HExPEC clusters by the presence of the *fec* gene operon (Table 1, gene profile D).

**TABLE 1:** Gene presence/absence profile for the discriminating the human ExPEC clusters HExPEC-1 and HExPEC-2 apart from the mixed A/E-PEC cluster

Prevalence (%)			Gene presence/absence profile^*a*^												Gene profile
A/E-PEC	HExPEC-1	HExPEC-2	*yjjW*	gene3477	*yaeP*	gene8134	*fecA*2	*fecB*	*fecC*	*fecD*	*fecE*	*fecI*	*cutC*	*cutD*	
98	0	98	−	−	+										**A**
0	98	0	+	+	−										**B**

0	96	6				+	−	−	−	−	−	−			**C**
92	2	2				−	**+**	**+**	**+**	**+**	**+**	**+**			**D**
0	0	94				−	−	−	−	−	−	−			**E**

0	0	98											**+**	**+**	**F**

a Open or blank gene cells were not relevant for the gene profile.

10.1128/mSphere.00333-18.3 TABLE S1 Putative gene sequences. Download Table S1, DOCX file, 0.01 MB. Copyright © 2019 Jørgensen et al. 2019 Jørgensen et al. This content is distributed under the terms of the Creative Commons Attribution 4.0 International license.

### FimH typing.

Subdivision by FimH typing revealed a correlation between the FimH variants and the individual branches in both the SNP phylogeny and accessory-genome-derived tree (Fig. S1A and B). The mixed branches of APEC and ExPEC belonging to A/E-PEC carried genes encoding multiple FimH variants, including FimH15, FimH27, FimH30, and FimH54, and APEC strains were represented among all these FimH variants. Three genomes encoded two novel FimH variants, FimH526 and FimH525, each differing only by one nucleotide from FimH15 and FimH30, respectively, and grouped together with these. All strains encoding the FimH41 variant belonged to the HExPEC-1 cluster. Furthermore, all but one strain encoding the FimH18 variant belonged to the HExPEC-2 cluster.

### ExPEC-associated genes.

All strains were investigated for 55 genes previously described to be distinct for ExPEC (Table 2). Interestingly, 10 genes of these were present in all or >95% of all investigated genomes. Six additional genes were also highly frequent and were present in >75% of all genomes. Conversely, 14 genes were not identified or found in <5% of the genomes.

**TABLE 2:** Prevalence of the investigated ExPEC-associated genes

Category and gene^*a*^	Description	Prevalence (%)					GenBank accession no.
		APEC (*n* = 32)	ExPEC (*n* = 291)	A/E-PEC (*n* = 127)	HExPEC-1 (*n* = 147)	HExPEC-2 (*n* = 49)	
A/E-PEC-associated genes							
*iucC*^1^	Aerobactin synthesis	96.9	35.4	85.4	14.5	4.2	AAS66995.1
*iucD*^1^	Aerobactin synthesis	96.9	35.4	86.2	13.8	4.2	AAA23196.1
*iutA*^1^	Iron transport	96.9	35.1	84.6	14.5	4.2	AAS66997.1
*cvaA*^1^	Colicin V	96.9	33.0	83.1	13.1	0.0	CAA11514.1
*etsA*^1^	ABC transport system	96.9	30.6	77.7	13.1	0.0	YP_444079.1
*hlyF*^1^	Hemolysin	93.8	34.4	85.4	13.1	0.0	AAO49613.1
*ompT*^1/2^	Outer membrane protease	93.8	34.0	84.6	13.1	0.0	ADK70174.1
*cvaB*^1^	Colicin V	87.5	33.3	81.5	13.1	0.0	CAA11515.1
*cvaC*^1^	Colicin V	84.4	32.3	78.5	13.1	0.0	CAA40746.1
*cvi*^1^	Colicin V immunity	84.4	33.3	80.8	13.1	0.0	CAA11513.1
							
HExPEC-1- and A/E-PEC- associated genes							
*tia*^2^	Invasion determinant protein	71.9	75.6	84.6	91.7	0.0	ABJ02397.1
*papE*^2^	P fimbria	71.9	75.3	83.8	91.7	0.0	CAA43568.1
*ireA*^2^	Siderphore receptor	71.9	74.9	83.1	91.7	0.0	AMR36194.1
*papG*-II^2^	P fimbria adhesin variant	71.9	74.9	83.8	91.0	0.0	AAA24293.1
*papA*^2^	P fimbria shaft	68.8	74.6	81.5	91.7	0.0	AAZ04426.1
							
HExPEC-2-associated genes							
*ibeA*^2^	Brain epithelium invasion	40.6	21.3	20.0	0.7	100	AAA92443.1
*sfaS*^2^	S fimbria adhesin	28.1	19.6	13.8	0.7	97.9	AAB25046.1
*sfaG*^2^	S fimbria	28.1	18.6	11.5	0.7	97.9	AAB25045.1
*cnf1*^2^	Cytotoxic necrotizing factor	0.0	17.2	3.1	0.7	93.8	CAA50007.1
*papG-*III^2^	P fimbria adhesin variant	0.0	15.8	0.8	0.0	93.8	WP_001468556.1
							
HExPEC-2- and A/E-PEC- associated gene							
*iroN*^1^	Siderophore	96.9	49.1	83.1	13.1	97.9	AAN76093.1
							
Highly frequent (genes present in >75% of all strains)							
*csgA*^1/2^	Curli	100	100	100	100	100	AAA23616.1
*fyuA*^2^	Siderophore	100	100	100	100	100	CAA86211.1
*ompA*^2^	Serum resistance	100	100	100	100	100	AAN79561.1
*iss*^1^	Serum survival	100	100	100	100	100	X52665
*feoB*^3^	Iron uptake	100	99.7	100	99.3	100	NP_417868.1
*fimH*^2^	Type 1 fimbria adhesin	100	99.7	100	99.3	100	CAA12423.1
*usp*^2^	Uropathogenic-specific protein (bacteriocin)	100	99.3	99.2	99.3	100	BAA93674.1
*irp2*^2^	Yersiniabactin	100	98.6	98.5	98.6	100	OAC27446.1
*kpsMT-*K1^2^	Group 2 capsule kpsM variant	100	99.7	99.2	100	100	AAA24046.1
*malX* (PAI)^2^	Pathogenicity island	81.3	98.3	91.5	100	100	AF003742.1
*papC*^2^	P fimbria usher	71.9	91.1	84.6	91.0	95.8	CAA43564.1
*papF*^2^	P fimbria subunit	71.9	91.4	85.4	91.7	93.8	AAZ04419.1
*vat*^2^	Vacuolating autotransporter toxin	59.4	88.0	80.0	96.6	64.6	AAO21903.1
*bor*^3^	Serum resistance	96.9	77.3	74.6	88.3	64.6	AGD80583.1
*sitA*^2^	Iron transport	53.1	78.0	54.6	88.3	93.8	CAJ21596.1
							
Genes of variable presence							
*traT*^1^	Serum resistance	43.8	57.4	50.0	66.9	39.6	AAA26075.1
*eitA*^1^	Iron ABC transport system	31.3	11.7	10.8	9.7	33.3	ABD51672.1
*cma*^1^	Colicin M	34.4	2.4	13.1	0.7	0.0	AFK30933.1
*cbi*^1^	Colicin B immunity	31.3	2.7	13.1	0.7	0.0	YP_003937726.1
*cdtB*^2^	Cytolethal distending toxin B	0.0	5.8	12.3	0.0	2.1	BAL47227.1
Low frequency (genes identified in <5% of all investigated genomes)							
*astA*^2^	EAST1 toxin	12.5	1.0	4.6	0.7	0.0	AAG18472.1
*tsh*^1^	Hemagglutinin	12.5	0.0	3.1	0.0	0.0	AAA24698.1
*iha*^1^	Nonfimbrial Iha adhesin	0.0	0.3	0.0	0.7	0.0	ABB17254.1
*focG*^2^	F1C fimbria	0.0	0.3	0.8	0.0	0.0	AAB20438.1
*afa*^2^	Nonfimbrial adhesin	0.0	0.0	0.0	0.0	0.0	CAA54113.1
*fliC-*H7^2^	Flagellin	0.0	0.0	0.0	0.0	0.0	AGA03821.1
*hlyD*^2^	α-Hemolysin	0.0	0.0	0.0	0.0	0.0	YP_002756568.1
*papG-*I^2^	P fimbria adhesin variant	0.0	0.0	0.0	0.0	0.0	CAA43570.1
*bfp*^1^	Bundle-forming pilus	0.0	0.0	0.0	0.0	0.0	AID54475.1
*bma*	Blood group M adhesin	0.0	0.0	0.0	0.0	0.0	AAA23523.1
*gafD*	G-fimbriae	0.0	0.0	0.0	0.0	0.0	AAA69514.1
*rfc*^2^	Lipopolysaccharide synthesis	0.0	0.0	0.0	0.0	0.0	AAC43898.1
*stgA*^2^	Stg fimbria	0.0	0.0	0.0	0.0	0.0	AAS99229.1
*stx*^2^	Shiga toxin	0.0	0.0	0.0	0.0	0.0	AB012101.1

a The category is the gene location or gene frequency class. Genes localized on plasmid are indicated by superscript 1, genes localized on chromosome are indicated by superscript 2, and genes carried on phage are indicated by superscript 3.

**FIG 2:**
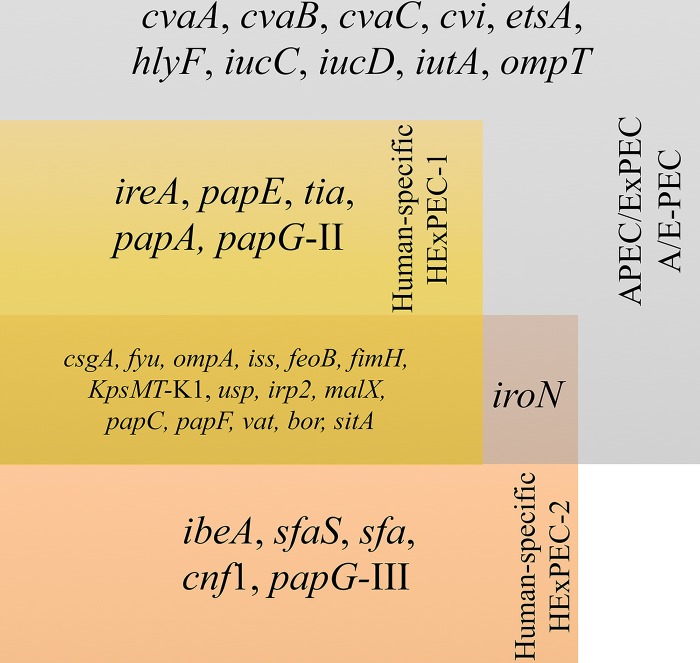
Diagram of ExPEC-associated gene distribution in the three groups; the two human ExPEC clusters HExPEC-1 and HExPEC-2 and the mixed avian/human ExPEC A/E-PEC cluster. Genes associated with the A/E-PEC are located in the gray box. HExPEC-1-associated genes are located in the yellow box. HExPEC-2- associated genes are located in the orange box.

A correlation was observed between the virulence-associated genes and the A/E-PEC group and the two HExPEC groups (Fig. 2). Ten genes (*cvaA*, *cvaB*, *cvaC*, *cvi*, *etsA*, *hlyF*, *iucC*, *iucD*, *iutA*, and *ompT*) were significantly more prevalent (*P* < 0.01) in the A/E-PEC compared to the strains from either of the two HExPEC clusters. Five genes (*ireA*, *papA*, *papE*, *papG*-II, and *tia*) were found in almost all and significantly more HExPEC-1 and A/E-PEC, while were absent in the strains belonging to HExPEC-2 (*P* < 0.001). Meanwhile, five genes (*ibeA*, *sfaS*, *sfa*, *cnf1*, and *papG*-III) were significantly more prevalent in HExPEC-2 (*P* < 0.001). The *iroN* gene was detected in significantly fewer HExPEC-1 strains compared to A/E-PEC and HExPEC-2 (*P* < 0.0001).

### Plasmids.

All genomes were investigated for virulence-associated plasmids using PlasmidSeeker (24). Multiple closely related variants of the pAPEC-O2-ColV, pAPEC-O1, p1ColV5155, and pAPEC-O78-ColV plasmid types were identified. All APEC strains harbored ColV plasmids, while only 25% of the human ExPEC strains carried plasmids. None of HExPEC-2 strains harbored ColV plasmids, while 11.7% of the HExPEC-1 strains contained the pAPEC-O78-ColV plasmid. Meanwhile, 70% of the human ExPEC strains belonging to the A/E-PEC clusters harbored ColV plasmids, corresponding to 78% of all A/E-PEC strains that contained plasmids.

### Antibiotic resistance.

The levels of antibiotic resistance were investigated *in silico* (Table 3). There was no statistical difference in the number of APEC and human ExPEC resistant strains for any of the classes of antibiotics. HExPEC-2 demonstrated the highest prevalence of beta-lactam, sulfonamide, trimethoprim, tetracycline, and macrolide antibiotic resistance genes. However, only the prevalence of beta-lactam and tetracycline genes were significantly higher in HExPEC-2 than in A/E-PEC (*P* < 0.05). Furthermore, significantly more A/E-PEC carried sulfonamide and aminoglycoside resistance genes than HExPEC-1 (*P* < 0.05). Strains with genes coding for ≥3 classes of antibiotic resistance genes were considered multiantibiotic resistant (Table 3). While there was no difference in the number of putative multidrug-resistant strains between the APEC and human ExPEC, there were a significantly higher number of multiantibiotic-resistant HExPEC-2 strains compared to both the HExPEC-1 and A/E-PEC (*P* < 0.01 and *P* < 0.05, respectively).

**TABLE 3:** Prevalence antibiotic classes of the different ExPEC subtypes

Antibiotic resistance class	Prevalence (%)					
	Total (*n* = 323)	APEC (*n* = 32)	Human ExPEC (*n* = 291)	A/E-PEC (*n* = 127)	HExPEC-1 (*n* = 147)	HExPEC-2 (*n* = 49)
Beta-lactams	18.3	6.3	19.6	14.6	17.9	29.2
Sulfonamides	16.1	12.5	16.5	20.0	9.0	27.1
Trimethoprims	8.7	3.1	9.3	9.2	4.8	18.8
Aminoglycosides	7.4	9.4	7.2	11.5	3.4	8.3
Tetracyclines	4.6	3.1	4.8	3.1	3.4	12.5
Macrolides	1.2	0.0	1.4	0.8	1.4	2.1
Quinolones	0.3	0.0	0.3	0.8	0.0	0.0

Multiresistance	11.0	6.3	11.0	10.0	6.9	22.9

## DISCUSSION

Previous studies have reported a genetic overlap in APEC and human ExPEC strains belonging to ST95 and suggested a zoonotic potential of APEC (21–23). However, these studies were solely based on comparing the APEC-O1 type strain to human ExPEC strains. Here, using whole-genome sequencing (WGS) data on a large collection of ST95 strains, APEC belonging to ST95 did not constitute a unique branch and were not associated with single SNPs or specific accessory genome features. The APEC genomes were generally diverse and located on multiple branches together with closely related human ExPEC, including nearly identical APEC and human ExPEC strains in both the SNP phylogeny and accessory-genome-derived trees. These results clearly show that APEC and human ExPEC belonging to ST95 could not be distinguished. The large overall genetic overlap and identification of dispersed clones of closely related APEC and human ExPEC support the previous findings and demonstrate that certain ST95 ExPECs do not exhibit host specificity or have host specificity against multiple hosts, including humans and birds. Furthermore, the genetic diversity between the investigated APEC genomes also reveal that no single ST95 APEC strain can be used as a ST95 APEC type strain to represent the entire ST95 APEC pathotype.

Two clusters consisted entirely of human ExPEC strains, the HExPEC clusters. There was a strong overrepresentation of strains collected in the USA in 2012 in the two HExPEC clusters compared to the mixed human ExPEC and APEC cluster, A/E-PEC (62% of HExPEC-1, 46% of HExPEC-2, and 24% of A/E-PEC) (*P* < 0.001), Thus, the clustering could be the result of skewed overrepresentation of clonally related strains. However, both HExPEC-1 and HExPEC-2 represented strains from multiple countries over a 67-year and 38-year period, HExPEC-1 and HExPEC-2, respectively, and resulted in closely related clusters in the SNP-based phylogenies. Though human ExPEC and APEC A/E-PEC genomes demonstrated a great genetic overlap suggesting low host association, these findings suggest that certain subclades of genetically stable ExPEC belonging to ST95 could have adapted a degree of host predilection.

The HExPEC-1 cluster was distinctly separated from the remaining genomes in both the SNP phylogeny and accessory-genome-derived tree, while the HExPEC-2 cluster demonstrated a clear separation only in the accessory-genome-derived tree. The HExPEC-1 was distinguishable from other strains by two canonical SNPs as well as several accessory genome features, while the HExPEC-2 cluster constituted a distinct subclade in the accessory genome, defined by the presence of two *cut* genes. The two canonical SNPs that differentiated the HExPEC-1 were both in the nicotinamide ribose kinase domain of the *nadR* gene and were both nonsynonymous. NadR is a trifunctional protein involved in regulation of NAD and associated fermentation pathways and anaerobic growth (25, 26). Two combinations of genes were found to be unique to the HExPEC-1. The first consisted of *yjjW*, *yaeP*, and gene3477 (Table 1, gene profile A/B), and the second consisted of gene8134 and the *fec* operon (Table 1, gene profile C/D/E). The YjjW protein has been associated with the activation of YjjI, a protein hypothesized to be involved in nitrogen metabolism. The *yaeO* gene has been suggested to be expressed through translational coupling with *yaeP* (27). YaeO has been shown to be a Rho-specific inhibitor of transcription termination, an essential process for the regulation of bacterial gene expression (28). The gene3477 gene encodes a putative transposase. The *fec* genes are signaling and transport genes for the binding and uptake of diferric dicitrate (29). Evidence indicates that *fec* genes are acquired by horizontal gene transfer and have been reported to be located in pathogenicity islands in Shigella flexneri. Meanwhile, gene8134 could not be annotated by Prokka, NCBI BLAST, or UniProt. For HExPEC-2, the presence of *cutC* and *cutD* genes was unique. The *cut* gene cluster has been described and associated with disease-associated microbial metabolic activity in humans and has been suggested to be horizontally transferred between bacteria in the human gut (30).

The prevalence of 55 genes, which previously have been suggested to demonstrate APEC or human ExPEC association were investigated (Table 2) (5, 9, 10). Interestingly, in this study, nine genes were found to be present in all investigated genomes (*csgA*, *fyu*, *ompA*, *iss*, *feoB*, *fimH*, *usp, kpsMT*-K1, and *irp2*). Additionally, six genes (*malX*, *papC*, *papF*, *vat*, *bor*, and *sitA*) were identified in >75% of all genomes and did not show any host or cluster association (Fig. 2). All investigated genomes originated from isolates from diseased birds or were clinical human isolates; thus, a degree of virulence potential in the strains was expected. However, several of the 15 frequently identified genes have previously been suggested to be host associated. The *iss*, *papC*, *vat,* and *sitA* genes have been suggested to be APEC associated, while *csgA* and *fimH* have been suggested to have stronger association with human ExPEC (5, 9, 10, 31). Additionally, *fyu* and *irp2* have been suggested to be associated with both host types. These results demonstrate that these frequently identified genes possibly have lower host association in ST95 ExPECs than previously predicted. Meanwhile, 14 of the 55 genes were not identified or found in very low numbers in the investigated strains, including the *astA*, *tsh*, *iha,* and *afa* genes which have been suggested to be host associated (9, 31) and generic for ExPEC; however, these genes appear not to be significant for ExPEC belonging to ST95.

The 10 A/E-PEC-associated genes (*cvaA*, *cvaB*, *cvaC*, *cvi*, *etsA*, *hlyF*, *iucC*, *iucD*, *iutA*, and *ompT*) were identified in the majority of included APEC isolates (84.4 to 96.6%) and seemed essential for the avian virulence potential (Table 2). These 10 genes were also identified in the majority (77.7 to 86.2%) of the human ExPEC strains belonging to the A/E-PEC clusters, supporting their close relatedness to APEC. Previous studies have described the function and pathogenic significance of the 10 A/E-PEC genes (5, 9, 10). Furthermore, the four ColV plasmids, pAPEC-O2-ColV, pAPEC-O1, p1ColV5155, and pAPEC-O78-ColV, and closely related variants have been described to be closely related, with large strongly conserved core genes, including the 10 A/E-PEC-associated genes (32–34). ColV plasmids were identified in all APEC and 70% of the human ExPEC strains belonging to the A/E-PEC clusters, corresponding to 78% of all A/E-PEC strains containing ColV plasmids. These results further underline the significance of these 10 genes and the ColV plasmids which have previously been described to be associated with pathogenicity (5, 9, 10, 32–34). Meanwhile, 11.7% of the HExPEC-1 strains were sporadically identified to harbor the pAPEC-O78-ColV plasmid. It is generally accepted that transconjugation of virulence-associated plasmids can induce the recipient’s virulence potential, and transmission of pAPEC-O2-ColV into commensal E. coli strains has been demonstrated to significantly increase the virulence potential in *in ovo* experiments (12). This suggests that the avian virulence potential can possibly be introduced into human clones by the uptake of such plasmids.

The identified *ireA, papE, tia, papA,* and *papG*-II virulence genes encoded by HExPEC-1 could be host species related or associated. However, the majority of the A/E-PEC also carried these genes, which emphasizes A/E-PEC’s potential to infect humans as well as birds. While few HExPEC-2 carried the A/E-PEC- and HExPEC-1-associated genes, five genes (*ibeA*, *sfaS*, *sfa*, *cnf1*, and *papG*-III) were significantly (*P* < 0.001) and almost exclusively found in the strains of the HExPEC-2 cluster compared to the A/E-PEC and HExPEC-1. These findings could indicate a lower virulence potential of HExPEC-2 strains toward birds, but an increase in the clinical virulence potential in a putative human subclade (Fig. 2). Interestingly, the *papG*-II variant was exclusively identified in the HExPEC-1 and A/E-PEC, while *papG*-III was almost only identified in the HExPEC-2. One may speculate that the P-fimbria adhesin variant PapG-II might have a significant role in the pathogenicity in both birds and humans, while one might suggest that the PapG-III variant had less avian pathogenicity potential.

Antimicrobial resistance genes toward the beta-lactam, sulfonamide, and trimethoprim classes were the most prevalent. Beta-lactams are among the most frequently used antimicrobial agents in both poultry and humans, which consequently can be reflected by high levels of beta-lactam resistance (35). Trimethoprim-sulfonamide combinations are broad-spectrum antimicrobial agents frequently used in poultry production as well as for treatment against urinary tract infection (UTI) (35). The prevalence of beta-lactam- and tetracycline-resistant HExPEC-2 strains was significant compared to that of A/E-PEC (*P* < 0.05), while A/E-PEC demonstrated significantly more sulfonamide- and aminoglycoside-resistant strains compared to HExPEC-1 (*P* < 0.05). The overall prevalence of antimicrobial resistance was observed in similar or lower levels as currently observed in poultry and clinical cases and did not show any strong correlation with any of the observed clusters (35). The aminoglycoside resistance gene *aac3Via* was the only gene found in significantly higher numbers of APEC compared to human ExPEC (*P* < 0.01) (see Fig. S1 in the supplemental material). Furthermore, the prevalence of *strA*, *strB*, *tetA*, *tetB*, *sul2*, *dfrA5*, and *aac3VI* genes were identified in significant higher numbers in A/E-PEC than in the HExPEC clusters. Tetracyclines are commonly used antimicrobials in poultry as well as in other animal species, which could explain the high prevalence of the *tetA* and *tetB* genes (35).

Typing of E. coli using concatenated FimH and MLST sequences has been suggested to have a greater discriminatory power than MLST alone (36). FimH typing correlated highly with the clustering of the accessory-derived tree, the presence of virulence-associated plasmids and additional virulence genes. APEC and human ExPEC strains belonging to A/E-PEC were found to have genes encoding FimH15/FimH526, FimH27, FimH30/FimH525, and FimH54 variants which correlated highly with the subclades of the A/E-PEC. While the strains encoding the FimH18 and FimH41 variants belonged to the HExPEC-2 and HExPEC-1, respectively, possibly this was due to the result of the strong overrepresentation of North American strains in the two clusters; the A/E-PEC-associated FimH groups were represented by temporally and spatially diverse strains (Table S2 and Fig. S1A and B).

10.1128/mSphere.00333-18.4 TABLE S2 List of strains used in this study. Download Table S2, DOCX file, 0.1 MB. Copyright © 2019 Jørgensen et al. 2019 Jørgensen et al. This content is distributed under the terms of the Creative Commons Attribution 4.0 International license.

While limited information is available on FimH15 and FimH54, previous studies have reported the FimH18, FimH27, FimH30, and FimH41 variants belonging to ST95 that are able to cause extraintestinal infections in humans (37, 38). One might suggest that combined MLST and FimH typing could give a greater overview of the subclades of ST95 E. coli strains capable of infecting both birds and humans.

In summary, APEC belonging to ST95 did not constitute a unique cluster, and these strains exhibited high genetic diversity. A large genetic overlap was observed between APEC and certain human ExPEC, including closely related clones. These results suggest a genotype that is not host specific and may be pathogenic in both birds and humans. Ten ExPEC genes were highly associated with avian pathogenicity. The genes were encoded on transferable plasmids, which were closely related to APEC-associated pAPEC-O2-ColV, pAPEC-O1, p1ColV5155, and pAPEC-O78-ColV. Additionally, aminoglycoside and sulfonamide resistance, including specific antimicrobial resistance-associated genes, tended to be more prevalent in A/E-PEC than in HExPEC.

Meanwhile, the large human exclusive ExPEC cluster, HExPEC-1, was identified by both SNP phylogeny and accessory-genome-derived tree, which represented a genetically stable human-specific clone. The additional HExPEC-2 cluster was identified in the accessory-genome-derived tree. Neither of the HExPEC-2 genomes harbored any of the virulence-associated plasmids related to avian hosts and carried fewer ExPEC-associated virulence genes in general. However, the geotemporal relationship of the strains of the HExPEC-1 and HExPEC-2 clusters suggests that these human ExPEC clusters do not necessarily reflect specific large human host-adapted genotypes but could merely be clonally related isolates from historical outbreak investigations. Though HExPEC might denote a lower virulence potential toward birds, transmission of virulence-associated genes carried on plasmids, including A/E-PEC-associated genes, are capable of introducing avian virulence potential as well as increasing the human virulence potential. The high genomic plasticity and rapid transmission of genes in E. coli further underline the challenges when defining host associations.

These findings suggest that multiple lineages of ExPEC belonging to ST95 exists, of which the majority may cause infection in humans, while only part of the ST95 cluster seem to be avian pathogenic. These results add s to a growing body of evidence that suggests that urinary tract infection may be a foodborne disease.

## MATERIALS AND METHODS

### Bacterial strains and genomes.

The genomes from 323 E. coli ExPEC strains belonging to the ST95 lineage were selected in accordance with the method of Johnson et al. (39) by carrying ≥2 ExPEC-associated genes. In total, 291 human ExPEC genomes and 32 APEC genomes were included in this study (see Table S2 in the supplemental material). A total of 146 genomes that fulfilled the criteria were obtained from EnteroBase (http://enterobase.warwick.ac.uk/species/index/ecoli), whereas 17 genomes were from GenBank. A total of 141 genomes were obtained from a 1-year prospective study in Flagstaff, Arizona. Seven genomes were obtained from the National Veterinary Institute, Technical University of Denmark. These seven isolates were previously included in another study, and the raw reads have been made available in the NCBI SRA under the study accession number SRP092633 (isolation IDs: E25, E28, E33, E37, E41, E42, and E43) (40). An additional 13 bacterial isolates were sequenced in-house, five E. coli isolates were from the University of Georgia, and eight isolates were from the strain collection at the Department of Veterinary and Animal Sciences, University of Copenhagen. The assemblies have been made available on NCBI under the BioProject accession number PRJNA431453. The 13 bacterial isolates were sequenced by 2x250-bp paired-end sequencing on a MiSeq instrument (Illumina) and assembled using SPAdes v3.5.0 (41). Initial analysis on all isolates was performed with MLST, PlasmidFinder, SerotypeFinder, FimTyper, and ResFinder (42–45).

### Analysis of the core genome.

Single nucleotide polymorphisms (SNPs) of the core genome were identified using NASP (46). All raw reads were aligned, and the sequence data of each genome were analyzed against the E. coli reference APEC O1 genome (GenBank accession no. CP000468). All positions with coverage less than 10× or if the variant was present in <90% of the base calls were excluded. For the univariate and multivariate analyses, all bases identical to the reference were set at “0,” while bases differing from the reference base were set at “1.” Dense areas of polymorphisms due to recombination or misalignment were removed using CleanRecomb by identification of stretches of consecutive sites with identical SNP profiles (47). An approximately maximum likelihood phylogeny was inferred using the FastTree 2.1.5 implementation in Geneious 9.1.8 (Biomatters, Auckland, New Zealand) on the purged SNPs. The tree was rooted using E. coli ST140 IMT5155 (GenBank accession ID CP005930) strain as an outgroup. All trees were visualized using iTol (48). Univariate analysis of the identified SNPs was performed by Fisher’s exact test. Significant *P* values (<0.05) were corrected for multiple testing using the false-discovery rate (FDR) method. Multivariate analysis of SNPs was performed by a discriminant analysis of principal components (DAPC) in R (version), using the *adegenet* package (49).

### Analysis of the accessory genome.

Open reading frames were identified and annotated with Prokka 1.12-beta with default settings, using barrnap 0.7 for rRNA prediction (50). Unannotated CDS were designated “geneXXXX.”

The pan-genome of annotated bacterial genomes was investigated using Roary 3.7.0 (51). The binary presence/absence data of accessory genes produced in Roary was used for the construction of an accessory binary tree as well as for univariate and multivariate analyses.

### ExPEC-associated genes and plasmid analysis.

The content of 55 previously described ExPEC-associated genes was investigated using BLASTn as implemented in Geneious 9.1.8 (10, 14, 21, 52). Reference genes were obtained from GenBank (Table 2). Genes were considered present with ≥85% coverage and ≥90% identity. Significant associations were analyzed using the Fisher exact test.

The genomes were investigated for large virulence-associated plasmids using PlasmidSeeker, with APEC-O1 as the reference (GenBank accession no. CP000468) (24). Identified plasmids with at least 80% Kmers found were considered a closely related variant of the respective plasmid.

10.1128/mSphere.00333-18.2 FIG S2 Percent abundance of antibiotic resistance genes for each of the groups; APEC, ExPEC, A/E-PEC, HExPEC-1, and HExPEC-2. Significant *P* values are indicated by asterisks as follows; *, *P* > 0.05; **, *P* > 0.01; ***, *P* > 0.001. Download FIG S2, TIF file, 0.1 MB. Copyright © 2019 Jørgensen et al. 2019 Jørgensen et al. This content is distributed under the terms of the Creative Commons Attribution 4.0 International license.
