# Supplementary figures and images for: Diversity and Population Overlap between Avian and Human Escherichia coli Belonging to Sequence Type 95
Source: mSphere. 2019 Jan 16;4(1):e00333-18. doi: 10.1128/mSphere.00333-18 (PMC6336079; doi:10.1128/mSphere.00333-18)

A

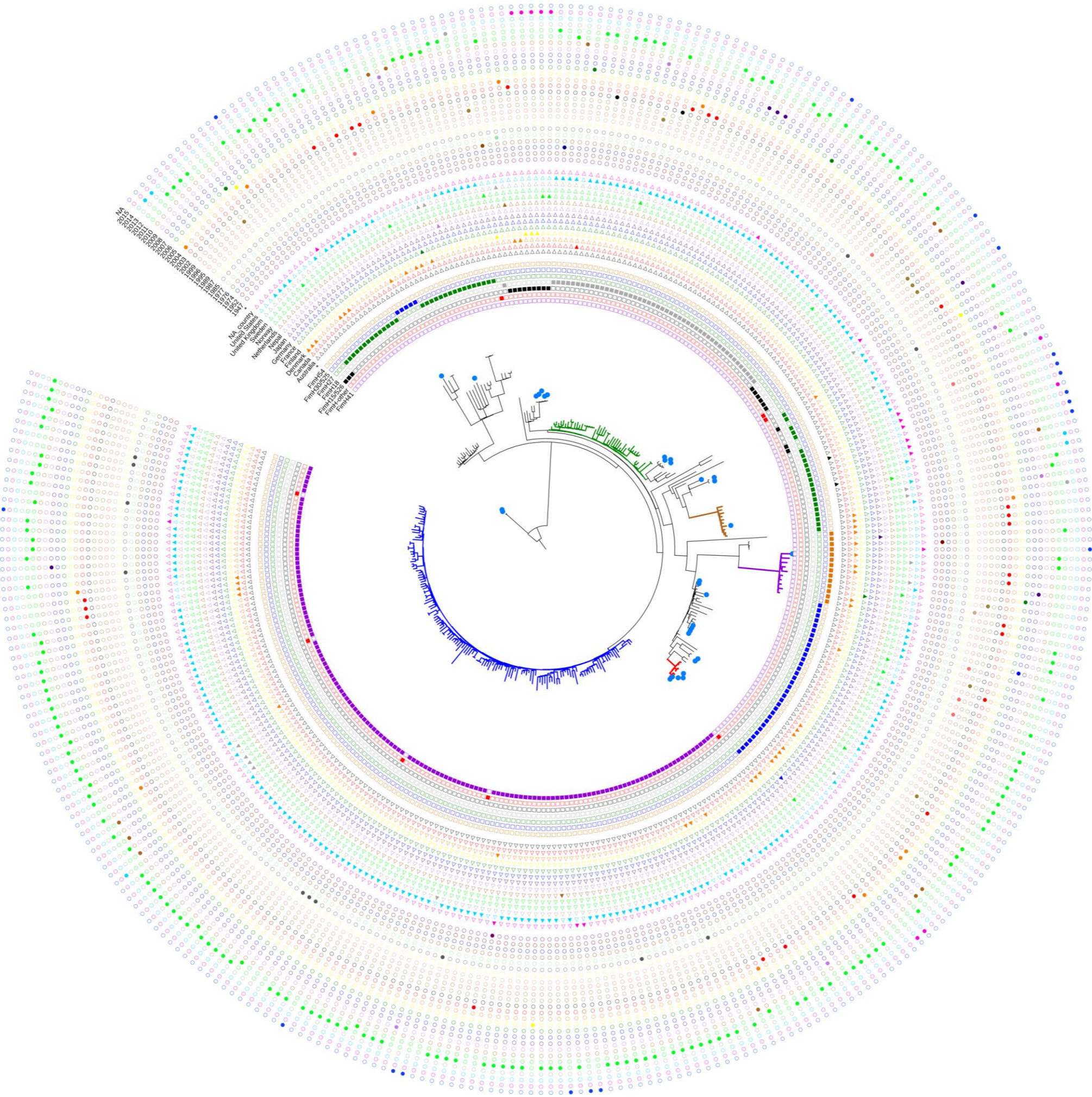

# B

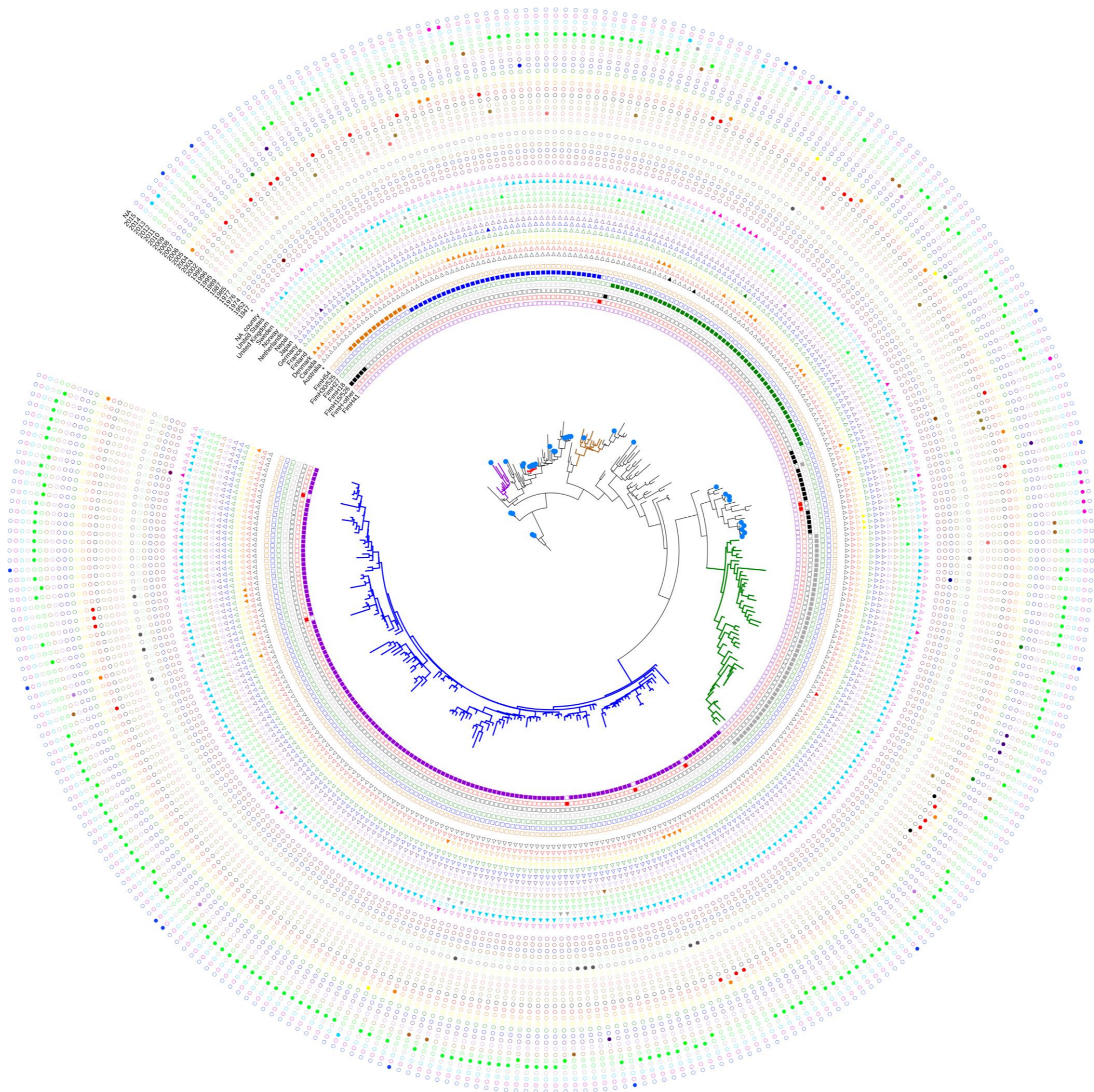

Supplement: FIG S1 [file mSphere.00333-18-sf001.pdf]

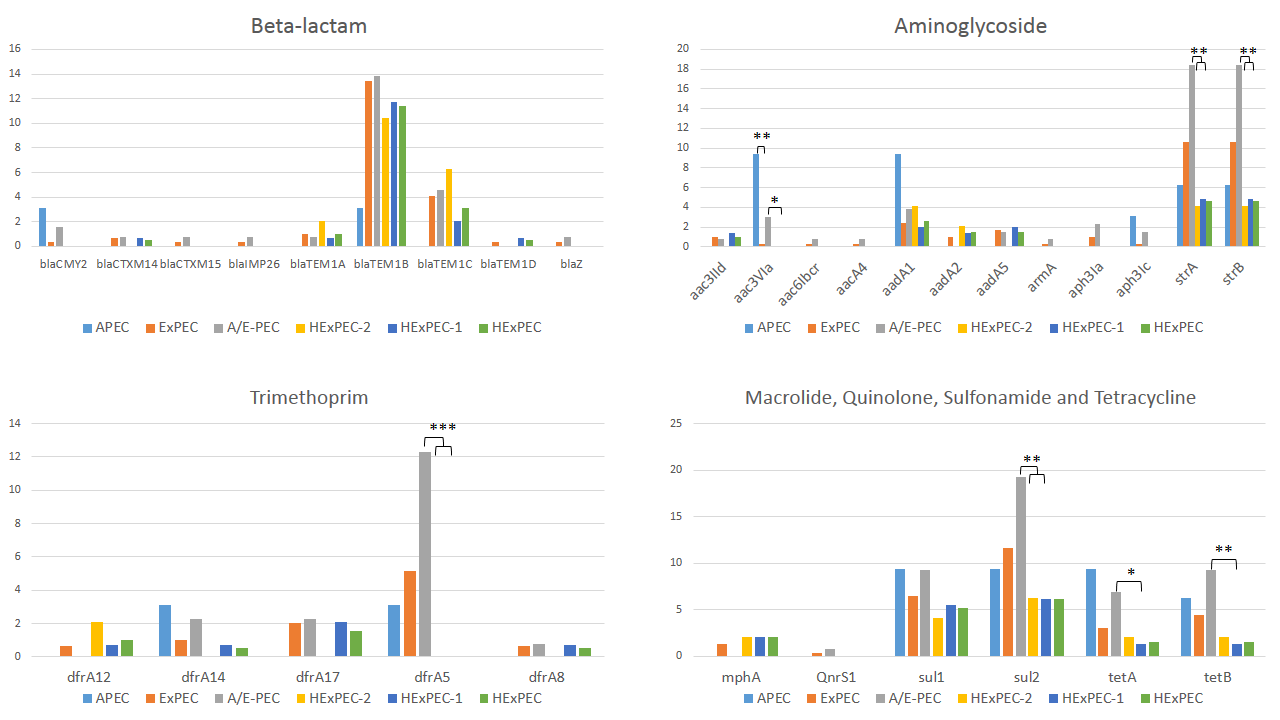

Supplement: FIG S2 [file mSphere.00333-18-sf002.tif]
