# Supplementary material for: Diversity and Population Overlap between Avian and Human Escherichia coli Belonging to Sequence Type 95
Source: mSphere. 2019 Jan 16;4(1):e00333-18. doi: 10.1128/mSphere.00333-18 (PMC6336079; doi:10.1128/mSphere.00333-18)
Supplement: TABLE S1 [file mSphere.00333-18-st001.docx]

| Gene 3477 | ATGGAACAAAAAACATTATCAGCTGAACCCCGCAGGTCATTTTCAAATGAGTTTAAACTTCAAATGGTTACACTGGCTTTACAACCAAGGGGTTCTGTTGCCCGTATTGCCCGGGAGCATGATATCAATGATAATCTTCTGTTCAAATGGCTCAGGCTCTCGCTGATCAAAGGGCGTATATCACGGCGTCTTCCGTTAACGAACTCTTCTGGCATTGGCGTTGAATTATTACCTGTGGAGATGACGCTGGGATGA |
| --- | --- |
| Gene8134 | GTGAACAACATCTTGAGAAAATCGCCAATCGAACGGCTTTGTTCATCTGTATCGATTACCCCTCATGAAATGGCTGTGGCCTTGGCTGGCCTAAACCCATCTATGCGCATTAGCGATGTGCCTGAGGAAAATTTCGAATATGTCGATTTTGTCAGAACTCATCTTGCCAGAGCTATCAAGGTGTACAGAGGTGAAAAAACTAGTAAAGACGAGCCTTGCCATGCCCTTGATATTTTCTTAGCTTCTTACCCATTTATTGACACAAATACACCAGAAATAATTGTTCAGAAAATATCAGAAGCGATTGATGATTTGCGTGGCACCAAGGGCTGGGAAGAAAAAGCCCGAAACCTTGGAGGACTCCAGTTAGTAAACTACATCAAAGAAACCAACCGCAGCGGACGTGGGCAGCATAGAAAGCAGGATGAGGAAAACGGGACAATGAAAATGATGGGGTTGATAGTACATCTGCTGATAAAAAAATCAGGCTCATCTTCCTACATCCAAAATGGGAAACCTAATCGCACAGCTATATACCGTGACATAGAAACCTTAATCAAGGATGAAGGGATATCCCATAAAGGAATAGCAAAAGCCACATTCCTAGAAAAAATCTCACGGGCTTTATTAGCTGTCCACGATCTCGACTAA |
