# Supplementary material for: Diversity and Population Overlap between Avian and Human Escherichia coli Belonging to Sequence Type 95
Source: mSphere. 2019 Jan 16;4(1):e00333-18. doi: 10.1128/mSphere.00333-18 (PMC6336079; doi:10.1128/mSphere.00333-18)
Supplement: TABLE S2 [file mSphere.00333-18-st002.docx]

| Name | Coli type | Host | Source | Country | Collection  Year | Genome sequence source |
| --- | --- | --- | --- | --- | --- | --- |
| 50857972 | ExPEC | Human | Human-Blood | Norway | 2014 | Enterobase |
| A192 | ExPEC | Human | Human-Blood | Netherlands | 1977 | Enterobase |
| AZ-TG73627 | APEC | Avian | Avian | United States | 2013 | Enterobase |
| AZ-TG73711 | APEC | Avian | Avian | United States | 2013 | Enterobase |
| BIDMC_49a | ExPEC | Human | Human-Blood | United States | 2012 | Enterobase |
| blood-08-0493 | ExPEC | Human | Human-Blood | United States | 2008 | Enterobase |
| blood-08-0654 | ExPEC | Human | Human-Blood | United States | 2008 | Enterobase |
| blood-09-0751 | ExPEC | Human | Human-Blood | United States | 2009 | Enterobase |
| blood-10-0686 | ExPEC | Human | Human-Blood | United States | 2010 | Enterobase |
| blood-10-0687 | ExPEC | Human | Human-Blood | United States | 2010 | Enterobase |
| blood-11-0031 | ExPEC | Human | Human-Blood | United States | 2011 | Enterobase |
| blood-11-0041 | ExPEC | Human | Human-Blood | United States | 2011 | Enterobase |
| BS11 | ExPEC | Human | Human-Blood | Australia | 2014 | Enterobase |
| BS14 | ExPEC | Human | Human-Blood | Australia | 2015 | Enterobase |
| C525 | ExPEC | Human | Human-Blood | Nepal | 2010 | Enterobase |
| GN02005 | ExPEC | Human | Human-Blood | United States | 2002 | Enterobase |
| GN02099 | ExPEC | Human | Human-Blood | United States | 2002 | Enterobase |
| GN02148 | ExPEC | Human | Human-Blood | United States | 2003 | Enterobase |
| GN02165 | ExPEC | Human | Human-Blood | United States | 2003 | Enterobase |
| GN02172 | ExPEC | Human | Human-Blood | United States | 2003 | Enterobase |
| GN02254 | ExPEC | Human | Human-Blood | United States | 2003 | Enterobase |
| GN02260 | ExPEC | Human | Human-Blood | United States | 2004 | Enterobase |
| GN02345 | ExPEC | Human | Human-Blood | United States | 2005 | Enterobase |
| GN02476 | ExPEC | Human | Human-Blood | United States | 2006 | Enterobase |
| GN03324 | ExPEC | Human | Human-Blood | United States | 2010 | Enterobase |
| GN03409 | ExPEC | Human | Human-Blood | United States | 2010 | Enterobase |
| GN04262 | ExPEC | Human | Human-Blood | United States | 2011 | Enterobase |
| GN04665 | ExPEC | Human | Human-Blood | United States | 2012 | Enterobase |
| GN04676 | ExPEC | Human | Human-Blood | United States | 2012 | Enterobase |
| GN05696 | ExPEC | Human | Human-Blood | United States | 2013 | Enterobase |
| HICF1 | ExPEC | Human | Human-Blood | United Kingdom | 2008 | Enterobase |
| HICF110 | ExPEC | Human | Human-Blood | United Kingdom | 2010 | Enterobase |
| HICF14 | ExPEC | Human | Human-Blood | United Kingdom | 2008 | Enterobase |
| HICF154 | ExPEC | Human | Human-Blood | United Kingdom | 2010 | Enterobase |
| HICF180 | ExPEC | Human | Human-Blood | United Kingdom | 2010 | Enterobase |
| HICF190 | ExPEC | Human | Human-Blood | United Kingdom | 2010 | Enterobase |
| HICF205 | ExPEC | Human | Human-Blood | United Kingdom | 2011 | Enterobase |
| HICF212 | ExPEC | Human | Human-Blood | United Kingdom | 2011 | Enterobase |
| HICF219 | ExPEC | Human | Human-Blood | United Kingdom | 2011 | Enterobase |
| HICF241 | ExPEC | Human | Human-Blood | United Kingdom | 2011 | Enterobase |
| HICF257 | ExPEC | Human | Human-Blood | United Kingdom | 2011 | Enterobase |
| HICF42 | ExPEC | Human | Human-Blood | United Kingdom | 2009 | Enterobase |
| HICF62 | ExPEC | Human | Human-Blood | United Kingdom | 2009 | Enterobase |
| HICF76 | ExPEC | Human | Human-Blood | United Kingdom | 2009 | Enterobase |
| HICF83 | ExPEC | Human | Human-Blood | United Kingdom | 2009 | Enterobase |
| HICF89 | ExPEC | Human | Human-Blood | United Kingdom | 2009 | Enterobase |
| HVH_1_4-6876161 | ExPEC | Human | Human-Blood | Denmark | 2004 | Enterobase |
| HVH_102_4-6906788 | ExPEC | Human | Human-Blood | Denmark | 2004 | Enterobase |
| HVH_104_4-6977960 | ExPEC | Human | Human-Blood | Denmark | 2004 | Enterobase |
| HVH_118_4-7345399 | ExPEC | Human | Human-Blood | Denmark | 2004 | Enterobase |
| HVH_12_4-7653042 | ExPEC | Human | Human-Blood | Denmark | 2005 | Enterobase |
| HVH_126_4-6034225 | ExPEC | Human | Human-Blood | Denmark | 2004 | Enterobase |
| HVH_127_4-7303629 | ExPEC | Human | Human-Blood | Denmark | 2004 | Enterobase |
| HVH_137_4-2124971 | ExPEC | Human | Human-Blood | Denmark | 2004 | Enterobase |
| HVH_148_4-3192490 | ExPEC | Human | Human-Blood | Denmark | 2004 | Enterobase |
| HVH_170_4-3026949 | ExPEC | Human | Human-Blood | Denmark | 2003 | Enterobase |
| HVH_178_4-3189163 | ExPEC | Human | Human-Blood | Denmark | 2003 | Enterobase |
| HVH_180_4-3051617 | ExPEC | Human | Human-Blood | Denmark | 2003 | Enterobase |
| HVH_19_4-7154984 | ExPEC | Human | Human-Blood | Denmark | 2005 | Enterobase |
| HVH_191_3-9341900 | ExPEC | Human | Human-Blood | Denmark | 2003 | Enterobase |
| HVH_192_4-3054470 | ExPEC | Human | Human-Blood | Denmark | 2003 | Enterobase |
| HVH_199_4-5670322 | ExPEC | Human | Human-Blood | Denmark | 2004 | Enterobase |
| HVH_201_4-4459431 | ExPEC | Human | Human-Blood | Denmark | 2003 | Enterobase |
| HVH_203_4-3126218 | ExPEC | Human | Human-Blood | Denmark | 2003 | Enterobase |
| HVH_210_4-3042480 | ExPEC | Human | Human-Blood | Denmark | 2003 | Enterobase |
| HVH_211_4-3041891 | ExPEC | Human | Human-Blood | Denmark | 2003 | Enterobase |
| HVH_214_4-3062198 | ExPEC | Human | Human-Blood | Denmark | 2003 | Enterobase |
| HVH_217_4-1022806 | UPEC | Human | Human-Urine | Denmark | 2003 | Enterobase |
| HVH_222_4-2977443 | ExPEC | Human | Human-Blood | Denmark | 2003 | Enterobase |
| HVH_3_4-7276001 | ExPEC | Human | Human-Blood | Denmark | 2004 | Enterobase |
| HVH_30_4-2661829 | ExPEC | Human | Human-Blood | Denmark | 2003 | Enterobase |
| HVH_32_4-3773988 | ExPEC | Human | Human-Blood | Denmark | 2003 | Enterobase |
| HVH_35_4-2962667 | ExPEC | Human | Human-Blood | Denmark | 2003 | Enterobase |
| HVH_42_4-2100061 | ExPEC | Human | Human-Blood | Denmark | 2003 | Enterobase |
| HVH_48_4-2658593 | ExPEC | Human | Human-Blood | Denmark | 2003 | Enterobase |
| HVH_5_4-7148410 | ExPEC | Human | Human-Blood | Denmark | 2004 | Enterobase |
| HVH_54_4-2723514 | ExPEC | Human | Human-Blood | Denmark | 2003 | Enterobase |
| HVH_59_4-1119338 | ExPEC | Human | Human-Blood | Denmark | 2003 | Enterobase |
| HVH_73_4-2393174 | ExPEC | Human | Human-Blood | Denmark | 2003 | Enterobase |
| HVH_76_4-2538717 | ExPEC | Human | Human-Blood | Denmark | 2003 | Enterobase |
| KOEGE_32_66a | UPEC | Human | Human-Urine | Denmark | 2006 | Enterobase |
| MOD1-EC5197 | ExPEC | Human | Human-Blood | United States | 1987 | Enterobase |
| MOD1-EC587 | ExPEC | Human | Human-Blood | NA | 1947 | Enterobase |
| MOD1-EC665 | ExPEC | Human | Human-Blood | United States | 1985 | Enterobase |
| MOD1-EC669 | ExPEC | Human | Human-Blood | United States | 1985 | Enterobase |
| MOD1-EC670 | ExPEC | Human | Human-Blood | United States | 1985 | Enterobase |
| MOD1-EC674 | ExPEC | Human | Human-Blood | United States | 1985 | Enterobase |
| MOD1-EC677 | ExPEC | Human | Human-Blood | United States | 1985 | Enterobase |
| MOD1-EC678 | ExPEC | Human | Human-Blood | United States | 1985 | Enterobase |
| MOD1-EC690 | ExPEC | Human | Human-Blood | United States | 1985 | Enterobase |
| MOD1-EC696 | ExPEC | Human | Human-Blood | United States | 1985 | Enterobase |
| MOD1-EC715 | ExPEC | Human | Human-Blood | United States | 1985 | Enterobase |
| MOD1-EC721 | ExPEC | Human | Human-Blood | United States | 1985 | Enterobase |
| MOD1-EC723 | ExPEC | Human | Human-Blood | United States | 1985 | Enterobase |
| MOD1-EC728 | ExPEC | Human | Human-Blood | United States | 1985 | Enterobase |
| PA10B | UPEC | Human | Human-Urine | NA | NA | Enterobase |
| PA11B | UPEC | Human | Human-Urine | NA | NA | Enterobase |
| PA28B | UPEC | Human | Human-Urine | NA | NA | Enterobase |
| PA41B | UPEC | Human | Human-Urine | NA | NA | Enterobase |
| PA45B | UPEC | Human | Human-Urine | NA | NA | Enterobase |
| PA47B | UPEC | Human | Human-Urine | NA | NA | Enterobase |
| PA50B | UPEC | Human | Human-Urine | NA | NA | Enterobase |
| PA63B | UPEC | Human | Human-Urine | NA | NA | Enterobase |
| PA70B | UPEC | Human | Human-Urine | NA | NA | Enterobase |
| PA72B | UPEC | Human | Human-Urine | NA | NA | Enterobase |
| PA8B | UPEC | Human | Human-Urine | NA | NA | Enterobase |
| SCP28-34 | ExPEC | Human | Human-Blood | Netherlands | NA | Enterobase |
| SF-088 | ExPEC | Human | Human-Blood | United States | 2007 | Enterobase |
| SF-166 | ExPEC | Human | Human-Blood | United States | 2008 | Enterobase |
| SF-173 | ExPEC | Human | Human-Blood | United States | 2008 | Enterobase |
| SF-468 | ExPEC | Human | Human-Blood | United States | 2010 | Enterobase |
| UMEA_3041-1 | UPEC | Human | Human-Urine | Sweden | 1996 | Enterobase |
| UMEA_3140-1 | UPEC | Human | Human-Urine | Sweden | 1995 | Enterobase |
| UMEA_3203-1 | UPEC | Human | Human-Urine | Sweden | 1995 | Enterobase |
| UMEA_3206-1 | UPEC | Human | Human-Urine | Sweden | 1995 | Enterobase |
| UMEA_3298-1 | UPEC | Human | Human-Urine | Sweden | 1995 | Enterobase |
| UMEA_3632-1 | UPEC | Human | Human-Urine | Sweden | 1996 | Enterobase |
| UMEA_3662-1 | UPEC | Human | Human-Urine | Sweden | 1996 | Enterobase |
| UMEA_3702-1 | UPEC | Human | Human-Urine | Sweden | 1996 | Enterobase |
| UMEA_3834-1 | UPEC | Human | Human-Urine | Sweden | 1996 | Enterobase |
| UMEA_3893-1 | UPEC | Human | Human-Urine | Sweden | 1996 | Enterobase |
| upec-106 | UPEC | Human | Human-Urine | United States | 2011 | Enterobase |
| upec-120 | UPEC | Human | Human-Urine | United States | 2011 | Enterobase |
| upec-124 | UPEC | Human | Human-Urine | United States | 2011 | Enterobase |
| upec-129 | UPEC | Human | Human-Urine | United States | 2011 | Enterobase |
| upec-131 | UPEC | Human | Human-Urine | United States | 2011 | Enterobase |
| upec-136 | UPEC | Human | Human-Urine | United States | 2011 | Enterobase |
| upec-139 | UPEC | Human | Human-Urine | United States | 2011 | Enterobase |
| upec-144 | UPEC | Human | Human-Urine | United States | 2011 | Enterobase |
| upec-157 | UPEC | Human | Human-Urine | United States | 2011 | Enterobase |
| upec-169 | UPEC | Human | Human-Urine | United States | 2011 | Enterobase |
| upec-185 | UPEC | Human | Human-Urine | United States | 2011 | Enterobase |
| upec-197 | UPEC | Human | Human-Urine | United States | 2011 | Enterobase |
| upec-209 | UPEC | Human | Human-Urine | United States | 2011 | Enterobase |
| upec-240 | UPEC | Human | Human-Urine | United States | 2011 | Enterobase |
| upec-249 | UPEC | Human | Human-Urine | United States | 2011 | Enterobase |
| upec-250 | UPEC | Human | Human-Urine | United States | 2012 | Enterobase |
| upec-255 | UPEC | Human | Human-Urine | United States | 2012 | Enterobase |
| upec-51 | UPEC | Human | Human-Urine | United States | 2011 | Enterobase |
| upec-61 | UPEC | Human | Human-Urine | United States | 2011 | Enterobase |
| upec-72 | UPEC | Human | Human-Urine | United States | 2011 | Enterobase |
| upec-73 | UPEC | Human | Human-Urine | United States | 2011 | Enterobase |
| upec-75 | UPEC | Human | Human-Urine | United States | 2011 | Enterobase |
| upec-76 | UPEC | Human | Human-Urine | United States | 2011 | Enterobase |
| upec-8 | UPEC | Human | Human-Urine | United States | 2011 | Enterobase |
| upec-94 | UPEC | Human | Human-Urine | United States | 2011 | Enterobase |
| upec-98 | UPEC | Human | Human-Urine | United States | 2011 | Enterobase |
| 1_CN-22_B1_M1_C1_P1 | APEC | Poultry | Poultry | United States | 2012 | George Washington University |
| 2_CN-22_B1_M1_C1_P2 | APEC | Poultry | Poultry | United States | 2012 | George Washington University |
| 5_CN-24_B18_M1_C1/2_P1 | APEC | Poultry | Poultry | United States | 2012 | George Washington University |
| 57_CN-11_B30_M1_C3_P1 | APEC | Poultry | Poultry | United States | 2012 | George Washington University |
| 58_CN-11_B30_M1_C3_P2 | APEC | Poultry | Poultry | United States | 2012 | George Washington University |
| 67_CN-12_B7_M1_C4_P1 | APEC | Poultry | Poultry | United States | 2012 | George Washington University |
| 68_CN-12_B7_M1_C4_P2 | APEC | Poultry | Poultry | United States | 2012 | George Washington University |
| UTI_01375 | UPEC | Human | Human-Urine | United States | 2012 | George Washington University |
| UTI_01397 | UPEC | Human | Human-Urine | United States | 2012 | George Washington University |
| UTI_01413 | UPEC | Human | Human-Urine | United States | 2012 | George Washington University |
| UTI_01415 | UPEC | Human | Human-Urine | United States | 2012 | George Washington University |
| UTI_01511 | UPEC | Human | Human-Urine | United States | 2012 | George Washington University |
| UTI_01521 | UPEC | Human | Human-Urine | United States | 2012 | George Washington University |
| UTI_01527 | UPEC | Human | Human-Urine | United States | 2012 | George Washington University |
| UTI_01530 | UPEC | Human | Human-Urine | United States | 2012 | George Washington University |
| UTI_01532 | UPEC | Human | Human-Urine | United States | 2012 | George Washington University |
| UTI_01573 | UPEC | Human | Human-Urine | United States | 2012 | George Washington University |
| UTI_01590 | UPEC | Human | Human-Urine | United States | 2012 | George Washington University |
| UTI_01598 | UPEC | Human | Human-Urine | United States | 2012 | George Washington University |
| UTI_01618 | UPEC | Human | Human-Urine | United States | 2012 | George Washington University |
| UTI_01619 | UPEC | Human | Human-Urine | United States | 2012 | George Washington University |
| UTI_01650 | UPEC | Human | Human-Urine | United States | 2012 | George Washington University |
| UTI_01659 | UPEC | Human | Human-Urine | United States | 2012 | George Washington University |
| UTI_01674 | UPEC | Human | Human-Urine | United States | 2012 | George Washington University |
| UTI_01679 | UPEC | Human | Human-Urine | United States | 2012 | George Washington University |
| UTI_01717 | UPEC | Human | Human-Urine | United States | 2012 | George Washington University |
| UTI_01718 | UPEC | Human | Human-Urine | United States | 2012 | George Washington University |
| UTI_01724 | UPEC | Human | Human-Urine | United States | 2012 | George Washington University |
| UTI_01729 | UPEC | Human | Human-Urine | United States | 2012 | George Washington University |
| UTI_01737 | UPEC | Human | Human-Urine | United States | 2012 | George Washington University |
| UTI_01740 | UPEC | Human | Human-Urine | United States | 2012 | George Washington University |
| UTI_01769 | UPEC | Human | Human-Urine | United States | 2012 | George Washington University |
| UTI_01771 | UPEC | Human | Human-Urine | United States | 2012 | George Washington University |
| UTI_01780 | UPEC | Human | Human-Urine | United States | 2012 | George Washington University |
| UTI_01781 | UPEC | Human | Human-Urine | United States | 2012 | George Washington University |
| UTI_01785 | UPEC | Human | Human-Urine | United States | 2012 | George Washington University |
| UTI_01856 | UPEC | Human | Human-Urine | United States | 2012 | George Washington University |
| UTI_01879 | UPEC | Human | Human-Urine | United States | 2012 | George Washington University |
| UTI_01898 | UPEC | Human | Human-Urine | United States | 2012 | George Washington University |
| UTI_01900 | UPEC | Human | Human-Urine | United States | 2012 | George Washington University |
| UTI_01906 | UPEC | Human | Human-Urine | United States | 2012 | George Washington University |
| UTI_01908 | UPEC | Human | Human-Urine | United States | 2012 | George Washington University |
| UTI_01911 | UPEC | Human | Human-Urine | United States | 2012 | George Washington University |
| UTI_01929 | UPEC | Human | Human-Urine | United States | 2012 | George Washington University |
| UTI_01956 | UPEC | Human | Human-Urine | United States | 2012 | George Washington University |
| UTI_01964 | UPEC | Human | Human-Urine | United States | 2012 | George Washington University |
| UTI_01968 | UPEC | Human | Human-Urine | United States | 2012 | George Washington University |
| UTI_01971 | UPEC | Human | Human-Urine | United States | 2012 | George Washington University |
| UTI_02001 | UPEC | Human | Human-Urine | United States | 2012 | George Washington University |
| UTI_02012 | UPEC | Human | Human-Urine | United States | 2012 | George Washington University |
| UTI_02027 | UPEC | Human | Human-Urine | United States | 2012 | George Washington University |
| UTI_02053 | UPEC | Human | Human-Urine | United States | 2012 | George Washington University |
| UTI_02058 | UPEC | Human | Human-Urine | United States | 2012 | George Washington University |
| UTI_02059 | UPEC | Human | Human-Urine | United States | 2012 | George Washington University |
| UTI_02092 | UPEC | Human | Human-Urine | United States | 2012 | George Washington University |
| UTI_02108 | UPEC | Human | Human-Urine | United States | 2012 | George Washington University |
| UTI_02120 | UPEC | Human | Human-Urine | United States | 2012 | George Washington University |
| UTI_02129 | UPEC | Human | Human-Urine | United States | 2012 | George Washington University |
| UTI_02165 | UPEC | Human | Human-Urine | United States | 2012 | George Washington University |
| UTI_02173 | UPEC | Human | Human-Urine | United States | 2012 | George Washington University |
| UTI_02180 | UPEC | Human | Human-Urine | United States | 2012 | George Washington University |
| UTI_02187 | UPEC | Human | Human-Urine | United States | 2012 | George Washington University |
| UTI_02200 | UPEC | Human | Human-Urine | United States | 2012 | George Washington University |
| UTI_02201 | UPEC | Human | Human-Urine | United States | 2012 | George Washington University |
| UTI_02203 | UPEC | Human | Human-Urine | United States | 2012 | George Washington University |
| UTI_02244 | UPEC | Human | Human-Urine | United States | 2012 | George Washington University |
| UTI_02253 | UPEC | Human | Human-Urine | United States | 2012 | George Washington University |
| UTI_02256 | UPEC | Human | Human-Urine | United States | 2012 | George Washington University |
| UTI_02257 | UPEC | Human | Human-Urine | United States | 2012 | George Washington University |
| UTI_02314 | UPEC | Human | Human-Urine | United States | 2012 | George Washington University |
| UTI_02331 | UPEC | Human | Human-Urine | United States | 2012 | George Washington University |
| UTI_02337 | UPEC | Human | Human-Urine | United States | 2012 | George Washington University |
| UTI_02343 | UPEC | Human | Human-Urine | United States | 2012 | George Washington University |
| UTI_02353 | UPEC | Human | Human-Urine | United States | 2012 | George Washington University |
| UTI_02385 | UPEC | Human | Human-Urine | United States | 2012 | George Washington University |
| UTI_02398 | UPEC | Human | Human-Urine | United States | 2012 | George Washington University |
| UTI_02426 | UPEC | Human | Human-Urine | United States | 2012 | George Washington University |
| UTI_02439 | UPEC | Human | Human-Urine | United States | 2012 | George Washington University |
| UTI_02446 | UPEC | Human | Human-Urine | United States | 2012 | George Washington University |
| UTI_02448 | UPEC | Human | Human-Urine | United States | 2012 | George Washington University |
| UTI_02475 | UPEC | Human | Human-Urine | United States | 2012 | George Washington University |
| UTI_02476 | UPEC | Human | Human-Urine | United States | 2012 | George Washington University |
| UTI_02478 | UPEC | Human | Human-Urine | United States | 2012 | George Washington University |
| UTI_02495 | UPEC | Human | Human-Urine | United States | 2012 | George Washington University |
| UTI_02500 | UPEC | Human | Human-Urine | United States | 2012 | George Washington University |
| UTI_02507 | UPEC | Human | Human-Urine | United States | 2012 | George Washington University |
| UTI_02509 | UPEC | Human | Human-Urine | United States | 2012 | George Washington University |
| UTI_02511 | UPEC | Human | Human-Urine | United States | 2012 | George Washington University |
| UTI_02523 | UPEC | Human | Human-Urine | United States | 2012 | George Washington University |
| UTI_02524 | UPEC | Human | Human-Urine | United States | 2012 | George Washington University |
| UTI_02539 | UPEC | Human | Human-Urine | United States | 2012 | George Washington University |
| UTI_02547 | UPEC | Human | Human-Urine | United States | 2012 | George Washington University |
| UTI_02566 | UPEC | Human | Human-Urine | United States | 2012 | George Washington University |
| UTI_02567 | UPEC | Human | Human-Urine | United States | 2012 | George Washington University |
| UTI_02592 | UPEC | Human | Human-Urine | United States | 2012 | George Washington University |
| UTI_02593 | UPEC | Human | Human-Urine | United States | 2012 | George Washington University |
| UTI_02610 | UPEC | Human | Human-Urine | United States | 2012 | George Washington University |
| UTI_02624 | UPEC | Human | Human-Urine | United States | 2012 | George Washington University |
| UTI_02633 | UPEC | Human | Human-Urine | United States | 2012 | George Washington University |
| UTI_02644 | UPEC | Human | Human-Urine | United States | 2012 | George Washington University |
| UTI_02646 | UPEC | Human | Human-Urine | United States | 2012 | George Washington University |
| UTI_02658 | UPEC | Human | Human-Urine | United States | 2012 | George Washington University |
| UTI_02660 | UPEC | Human | Human-Urine | United States | 2012 | George Washington University |
| UTI_02669 | UPEC | Human | Human-Urine | United States | 2012 | George Washington University |
| UTI_02673 | UPEC | Human | Human-Urine | United States | 2012 | George Washington University |
| UTI_02676 | UPEC | Human | Human-Urine | United States | 2012 | George Washington University |
| UTI_02682 | UPEC | Human | Human-Urine | United States | 2012 | George Washington University |
| UTI_02686 | UPEC | Human | Human-Urine | United States | 2012 | George Washington University |
| UTI_02688 | UPEC | Human | Human-Urine | United States | 2012 | George Washington University |
| UTI_02694 | UPEC | Human | Human-Urine | United States | 2012 | George Washington University |
| UTI_02697 | UPEC | Human | Human-Urine | United States | 2012 | George Washington University |
| UTI_02704 | UPEC | Human | Human-Urine | United States | 2012 | George Washington University |
| UTI_02738 | UPEC | Human | Human-Urine | United States | 2012 | George Washington University |
| UTI_02747 | UPEC | Human | Human-Urine | United States | 2012 | George Washington University |
| UTI_02752 | UPEC | Human | Human-Urine | United States | 2012 | George Washington University |
| UTI_02806 | UPEC | Human | Human-Urine | United States | 2012 | George Washington University |
| UTI_02817 | UPEC | Human | Human-Urine | United States | 2012 | George Washington University |
| UTI_02830 | UPEC | Human | Human-Urine | United States | 2012 | George Washington University |
| UTI_02839 | UPEC | Human | Human-Urine | United States | 2012 | George Washington University |
| UTI_02844 | UPEC | Human | Human-Urine | United States | 2012 | George Washington University |
| UTI_02850 | UPEC | Human | Human-Urine | United States | 2012 | George Washington University |
| UTI_02851 | UPEC | Human | Human-Urine | United States | 2012 | George Washington University |
| UTI_02867 | UPEC | Human | Human-Urine | United States | 2012 | George Washington University |
| UTI_02874 | UPEC | Human | Human-Urine | United States | 2012 | George Washington University |
| UTI_02877 | UPEC | Human | Human-Urine | United States | 2012 | George Washington University |
| UTI_02881 | UPEC | Human | Human-Urine | United States | 2012 | George Washington University |
| UTI_02886 | UPEC | Human | Human-Urine | United States | 2012 | George Washington University |
| UTI_02937 | UPEC | Human | Human-Urine | United States | 2012 | George Washington University |
| UTI_02947 | UPEC | Human | Human-Urine | United States | 2012 | George Washington University |
| UTI_02954 | UPEC | Human | Human-Urine | United States | 2012 | George Washington University |
| UTI_02959 | UPEC | Human | Human-Urine | United States | 2012 | George Washington University |
| UTI_02966 | UPEC | Human | Human-Urine | United States | 2012 | George Washington University |
| UTI_02969 | UPEC | Human | Human-Urine | United States | 2012 | George Washington University |
| UTI_02979 | UPEC | Human | Human-Urine | United States | 2012 | George Washington University |
| UTI_02993 | UPEC | Human | Human-Urine | United States | 2012 | George Washington University |
| UTI_03000 | UPEC | Human | Human-Urine | United States | 2012 | George Washington University |
| UTI_03015 | UPEC | Human | Human-Urine | United States | 2012 | George Washington University |
| UTI_03059 | UPEC | Human | Human-Urine | United States | 2012 | George Washington University |
| UTI_03079 | UPEC | Human | Human-Urine | United States | 2012 | George Washington University |
| UTI_03094 | UPEC | Human | Human-Urine | United States | 2012 | George Washington University |
| E25 | APEC | Broiler parent | Broiler parent | Norway | 2015 | National Veterinary Institute,  Technical University of Denmark |
| E28 | APEC | Broiler parent | Broiler parent | Norway | 2015 | National Veterinary Institute,  Technical University of Denmark |
| E33 | APEC | Broiler parent | Broiler parent | Norway | 2015 | National Veterinary Institute,  Technical University of Denmark |
| E37 | APEC | Broiler | Broiler | Denmark | 2015 | National Veterinary Institute,  Technical University of Denmark |
| E41 | APEC | Broiler parent | Broiler parent | Finland | 2015 | National Veterinary Institute,  Technical University of Denmark |
| E42 | APEC | Broiler parent | Broiler parent | Finland | 2015 | National Veterinary Institute,  Technical University of Denmark |
| E43 | APEC | Broiler parent | Broiler parent | Finland | 2015 | National Veterinary Institute,  Technical University of Denmark |
| DSM_30083 | UPEC | Human | Human-Urine | Denmark | 1952 | NCBI |
| GN02487 | ExPEC | Human | Human-Blood | United States | NA | NCBI |
| GN02609 | ExPEC | Human | Human-Blood | United States | NA | NCBI |
| GN02627 | ExPEC | Human | Human-Blood | United States | NA | NCBI |
| IHE3034 | NMEC | Human | Human | Finland | 1976 | NCBI |
| JCM20135 | ExPEC | Human | Human | Japan | 2012 | NCBI |
| NCTC9001 | UPEC | Human | Human-Urine | NA | NA | NCBI |
| O1 | APEC | Avian | Avian | United States | NA | NCBI |
| O18 | APEC | Avian | Avian | United States | 2015 | NCBI |
| pmv-1 | ExPEC | Human | Human | NA | NA | NCBI |
| RS218 | NMEC | Human | Human | United States | 1974 | NCBI |
| S88 | NMEC | Human | Human | France | 1989 | NCBI |
| UCD-JA38 | ExPEC | Human | Human-Blood | United States | 2014 | NCBI |
| UM141 | ExPEC | Human | Human | Canada | 2006 | NCBI |
| UTI89 | UPEC | Human | Human-Urine | United States | 2006 | NCBI |
| 3-14 | APEC | Avian | Avian | Denmark | 2014 | University of Copenhagen strain collection |
| 4-7 | APEC | Avian | Avian | Denmark | 2014 | University of Copenhagen strain collection |
| 8F | APEC | Avian | Avian | France | 2012 | University of Copenhagen strain collection |
| 96T | APEC | Broiler parent | Broiler parent | Germany | 2012 | University of Copenhagen strain collection |
| Cp4L37 | APEC | Layer | Layer | Denmark | 2011 | University of Copenhagen strain collection |
| Cp6Salp3 | APEC | Layer | Layer | Denmark | 2011 | University of Copenhagen strain collection |
| Cp6Salp8 | APEC | Layer | Layer | Denmark | 2011 | University of Copenhagen strain collection |
| H1S10 | APEC | Broiler parent | Broiler parent | Denmark | 2010 | University of Copenhagen strain collection |
| H2S5 | APEC | Broiler breeder | Broiler parent | Denmark | 2010 | University of Copenhagen strain collection |
| A351 | APEC | Poultry | Poultry | United States | NA | University of Georgia strain collection |
| A359 | APEC | Poultry | Poultry | United States | NA | University of Georgia strain collection |
| A369 | APEC | Poultry | Poultry | United States | NA | University of Georgia strain collection |
| A52 | APEC | Poultry | Poultry | United States | NA | University of Georgia strain collection |
| A92 | APEC | Poultry | Poultry | United States | NA | University of Georgia strain collection |
|  |  |  |  |  |  |  |
|  |  |  |  |  |  |  |
|  |  |  |  |  |  |  |
|  |  |  |  |  |  |  |
|  |  |  |  |  |  |  |
|  |  |  |  |  |  |  |
|  |  |  |  |  |  |  |
